# A case of purpura fulminans caused by *Hemophilus influenzae* complicated by reversible cardiomyopathy

**DOI:** 10.1186/2052-0492-2-13

**Published:** 2014-02-18

**Authors:** Akira Endo, Atsushi Shiraishi, Junichi Aiboshi, Yoshiro Hayashi, Yasuhiro Otomo

**Affiliations:** Trauma and Acute Critical Care Medical Center, Medical Hospital of Tokyo Medical and Dental University, 1-5-45 Yushima, Bunkyo-ku, Tokyo, 113-8519 Japan; Department of Intensive Care Medicine, Kameda Medical Center, Kamogawa, Chiba, 296-8602 Japan; UQ Centre for Clinical Research, The University of Queensland, Herston, Brisbane, St Lucia Queensland, 4072 Australia

**Keywords:** Amputation, Disseminated intravascular coagulation, Symmetric peripheral gangrene

## Abstract

Here, we report a case of a 41-year-old male diagnosed as septic shock with purpura fulminans (PF) infection. The causative organism was β-lactamase-negative ampicillin-resistant *Hemophilus influenzae*. He developed fulminant cardiac dysfunction approximately 1 h after admission, and the cause was considered to be septic cardiomyopathy. Blood pressure and oxygenation were maintained at adequate levels with the aid of extracorporeal membrane oxygenation (ECMO). The cardiac dysfunction was reversible, and he was successfully weaned from ECMO on day 12 of hospitalization. However, he needed amputation for all extremities because the infection spread to his limbs and eventually, succumbed to sepsis caused by empyema on day 34 of hospitalization. To the best of our knowledge, this is only the second case of PF caused by *H. influenzae* in an adult to be reported worldwide.

## Background

Purpura fulminans (PF) was first described in 1886 and characterized by the apparent shock and rapidly progressive symmetric peripheral gangrene caused by disseminated intravascular coagulation [1]. Mortality of PF patients is high (approximately 43%), and most survivors require amputation of the affected extremities [2]. *Neisseria meningitidis* and *Streptococcus pneumoniae* are typical causative organisms of PF [3]. However, *Hemophilus influenza* is also a potential cause of the disease. The delay in approval of the vaccine for *H. influenzae* type b (Hib) and the emergence of various drug-resistant strains became a concerning situation in Japan with regard to Hib infection, which can be complicated by meningitis, endophthalmitis, renal failure, and necrosis of the extremities. Here, we report a case of 41-year-old male who succumbed to PF due to invasive *H. influenzae* infection complicated by severe cardiomyopathy requiring extracorporeal membrane oxygenation (ECMO).

## Case presentation

### Case report

A 41-year-old man presented to the emergency room with complaints of fever, diarrhea, and fatigue for 24 h.

On examination, he was restless and his systolic blood pressure, pulse, respiratory rate, and temperature were 50 mmHg, 143 beats/min, 30 breaths/min, and 38.2°C, respectively. Oxygen saturation could not be recorded because of peripheral circulatory failure. Marked cyanosis was evident in the facial region, peripheral limbs, and penis. Lung auscultation revealed coarse breathing sounds.

Arterial blood gas analysis was suggestive of remarkable lactic acidosis with a serum lactate level of 11.5 mmol/L. His white blood cell and platelet counts were low at 1,600/μL and 36,000/μL, respectively. Serum urea nitrogen (36.1 mg/dL), creatinine (4.8 mg/dL), and C-reactive protein (25.6 g/dL) levels were elevated. Coagulation was prolonged (prothrombin time, 50.9% of normal), and fibrin/fibrinogen degradation product levels were higher (98.9 μg/dL). Furthermore, his procalcitonin and endotoxin levels were markedly elevated (457 ng/mL and 548 pg/mL, respectively).

Computed tomography (CT) revealed left-sided sinusitis, bilateral lung consolidation, and an increased bilateral concentration of perirenal fat tissue. Lesions requiring debridement or drainage were not evident. His spleen was small, with a size of 38 cm^3^ on CT.

The observed cyanosis rapidly developed into purpura within 1 h after arrival. Samples of blood, sputum, stool, and urine were obtained for microbiological evaluation, and imipenem/cilastatin (IPM/CS) was administered as empirical therapy (0.5 g q12h). His blood pressure continued to decline despite the infusion of large amounts of fluid and catecholamines. His carotid pulse was nonpalpable 1 h after the arrival. Recovery of spontaneous circulation was achieved after 2 min of resuscitation. Narrow QRS sinus tachycardia without ST-T changes was apparent on electrocardiography. Decision to introduce veno-arterial ECMO (V-A ECMO) was made because of prolonged hemodynamic catastrophe.

Approximately 12 h after admission, his pulse pressure was undetectable and no cardiac contraction could be discerned by ultrasonography. However, we maintained blood pressure and oxygenation at adequate levels with the aid of V-A ECMO. PMX therapy was induced at emergency room for elevated endotoxin level, but it was not effective because there was no improvement of vital signs in this case.

Three days after hospitalization, cardiac function began to improve, and the ejection fraction improved to 40% by day 5. However, poor lung oxygenation persisted (PaO_2_/FiO_2_ ratio was 50). To maintain oxygenation of the brain and coronary arteries, we changed the support protocol from V-A ECMO to veno-venous ECMO (V-V ECMO).

Notably, the creatine kinase (CK) MB levels were 261 IU/mL on day 1 of hospitalization; these levels gradually increased to a peak of 417 IU/mL on day 3 before gradually decreasing. These variations and the elevated CK levels influenced the CK-MB/CK ratio, which was 1%–2% during the hospital stay.

A diagnosis of PF due to invasive *H. influenzae* infection was made on the basis of the isolation of β-lactamase-negative ampicillin-resistant (BLNAR) *H. influenzae* from the patient's sputum and blood. Minimum inhibitory concentration values for antibiotics were as follows: ampicillin, 2 μg/mL; cefaclor, 16 μg/mL; cefotiam, 4 μg/mL; cefpodoxime proxetil, 8 μg/mL; and cefozopran, 16 μg/mL.

**Figure 1:**
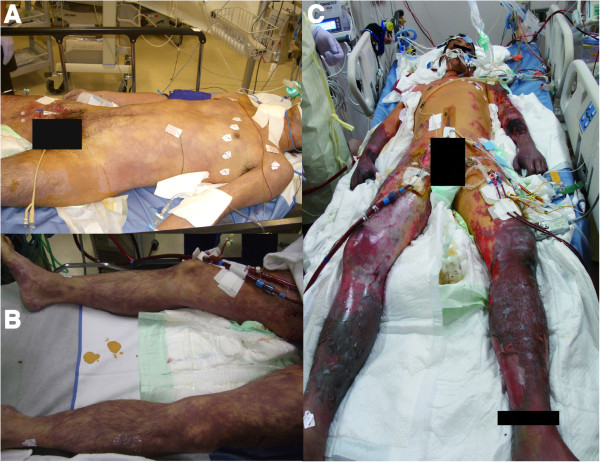
**Images of the patient was taken on the day 1 (A, B) and on the day 9 (C) of hospitalization.** Gangrene of the bilateral limbs, the facial region, and penis progressed gradually, and all limbs complicated with infection on the day 9.

On day 9 of hospitalization, infection-induced necrotic tissue in the limbs was clinically suspected (Figure 1), necessitating bilateral above-knee amputation and right forearm and left upper arm amputation. As the necrotic areas were not clearly demarcated, the stumps were not closed and daily debridement and irrigation was performed.

The patient's respiratory condition gradually improved thereafter, and he was successfully weaned from V-V ECMO on day 12 of hospitalization. However, on the same day, the patient developed left pneumothorax complicated by persistent air leakage, which resulted in empyema caused by methicillin-resistant *Staphylococcus aureus*. He improved enough to leave the catecholamines once, but septic shock due to empyema was rekindled. As oxygenation was too bad to perform isolated lung perfusion, surgical lavage and drainage were impossible. Daily lavage from a chest tube was performed, but he eventually succumbed to sepsis caused by the empyema on day 34 of hospitalization.

## Discussion

Here, we reported a case of PF caused by *H. influenzae* in a previously healthy adult. We conducted a literature search and found that approximately 20 pediatric cases and only one adult case have been reported [4]. The adult case of PF caused by *H. influenzae* was reported in 2006 and involved a 34-year-old man. Although he required bilateral lower limb and fingertip amputation, he managed to survive. As per our knowledge, our patient may be only the second such case reported worldwide.

The pathophysiology of PF is ischemia caused by disseminated intravascular coagulation (DIC). Though coagulation factor replacement strategies have been tested in several clinical trials, there is no established therapy for DIC. There have been reports that the congenital or secondary decrease of protein C are involved in the pathogenesis of PF. Additionally, there are reports of effective administration of activated protein C, which has not been approved in Japan [5]. The administration of 8 to 12 mL/kg of fresh frozen plasma (FFP) is effective for PF and protein C supplementation [6]. In the present case, 24 mL/kg of FFP was administered on the first day, but there was no obvious improvement. Recombinant thrombomodulin (rTM), which binds to thrombin *in vivo* and controls the ability to activate platelet function and fibrin formation, has been approved for DIC associated with sepsis in Japan. Moreover, there is a case report of limb salvage upon administration of rTM [7]. In our case, ECMO was introduced for a significant cardiac dysfunction, so rTM could not be used due to the high risk of bleeding.

Risks of this disease include hypoplasia of the spleen and post-splenectomy. In this case, splenic volume was 38 cm^3^ on CT, and the average splenic volume in adults is 131 cm^3^[8]. However, splenic volume is difficult to judge, because of large individual differences and changes at any given time in the same individual. In the present case, Howell-Jolly bodies were not observed in the hemogram.

Hib vaccination was initiated in the 1980s, and the incidence of invasive hemophilus infection has greatly decreased since then, with the added benefit of herd effects [5]. In Japan, however, Hib vaccination was not initiated until December 2008. Furthermore, the emergence of drug-resistant *H. influenzae* is a concerning issue in Japan, with up to 37.8% strains identified to be BLNAR [9]. In our patient, IPM/CS was administered as initial empirical therapy; however, the antibacterial effect of IPM/CS against *H. influenzae* is thought to be weak compared with that of other carbapenems [10]. Therefore, a different regime could have been used for managing the invasive hemophilus infection in our patient.

The surgical procedure and its timing are still controversial. Lerolle et al. recently reported the presence of bacteria in the damaged vascular wall of necrotic tissues [11]. However, previous literatures suggested that the main cause of necrosis is ischemia due to sepsis-induced DIC because of no bacteria in the necrotic tissues of PF. In addition, Johansen et al. did not recommend early amputation because the necrotic area of PF is gradually localized [12]. We planned the first operation after demarcating the necrotic area. However, necrotic tissues became to be infectious, so we were forced to perform amputations as a source control of infection. There are some reports about limb salvage managed by debridement and negative pressure wound therapy to the limited necrotic area caused by PF. In this case, intraoperative findings showed that muscles were extensively devastated. The height of amputation was determined by the muscle color and ability of contraction, and signs of infection.

To the best of our knowledge, there have been no other reports of severe cardiac dysfunction associated with PF. Several etiologies could have been responsible for the cardiac dysfunction in our patient. Stunned myocardium, bacterial myocarditis, and stress-induced cardiomyopathy are unlikely as the pathogenesis of cardiac dysfunction in this case. Because he did not show relevant changes of electrocardiography, pericardial effusion, or elevated cardiac enzyme levels, we assume that the possibility of septic cardiomyopathy is most probable in this case. Septic cardiomyopathy is characterized by defects in both the structure and function of the heart as a result of myocardial edema. Such defects include mitochondrial dysfunction, the collapse of myocardial microstructures, or both. These problems are associated with increased vascular permeability and elevated inflammatory cytokine levels [13]. Symptoms are evident even in the early stages of sepsis and are reversible. Septic cardiomyopathy is characterized by a decrease in ejection fraction attributable to diffuse hypokinesis of the left ventricular wall and a compensatory increase in the end-diastolic volume of the left ventricle. Cardiac index typically rises as a compensatory mechanism.

Several studies in animals have found that excessive levels of certain cytokines, including tumor necrosis factor-α, and interleukin-1β, induce cardiac dysfunction. Excessive nitric oxide and calcium levels are also reported to contribute to the development of such dysfunction, but these findings require corroboration [13, 14]. Recovery of cardiac function occurs over approximately 10 days in patients who survive an episode of septic cardiomyopathy. Our patient exhibited temporal worsening followed by a recovery in cardiac function; this is usually expected in patients with septic cardiomyopathy. However, he never displayed hyperdynamic state of typical sepsis development. Although our patient was atypical, we suggest that his condition was due to the failure of compensatory mechanisms related to septic cardiomyopathy.

Recently, usefulness of V-A ECMO has been reported in adult patients with sepsis [15]. In our case, ECMO had some curative effect for septic cardiomyopathy since the patient was not lost in the emergency room and could withstand the therapy for infectious disease. However, an overall evaluation of ECMO for septic cardiomyopathy is void because the patient eventually died.

## Conclusion

We reported the case of an adult male patient, possibly the second reported adult case till date, who developed PF caused by *H. influenzae*. His condition was complicated by reversible cardiomyopathy. The initial management with V-A ECMO was effective for early hemodynamic catastrophe, but he eventually succumbed to sepsis with empyema.

## Consent

Written informed consent was obtained from the family of the patient for the publication of this case report and any accompanying images. A copy of the written consent is available for review by the Editor-in-Chief of this journal.

## Abbreviations

PF: purpura fulminans
Hib: *H. influenzae* type b
ECMO: extracorporeal membrane oxygenation
CT: computed tomography
IPM/CS: imipenem/cilastatin
V-A ECMO: veno-arterial extracorporeal membrane oxygenation
V-V ECMO: veno-venous extracorporeal membrane oxygenation
CK: creatine kinase
BLNAR: β-lactamase-negative ampicillin-resistant
DIC: disseminated intravascular coagulation
FFP: fresh frozen plasma
rTM: recombinant thrombomodulin
